# DDX3 as a strongest prognosis marker and its downregulation promotes metastasis in colorectal cancer

**DOI:** 10.18632/oncotarget.4329

**Published:** 2015-06-02

**Authors:** Chia-Yi Su, Tsung-Chieh Lin, Yuan-Feng Lin, Ming-Huang Chen, Chien-Hsin Lee, Hsuan-Yao Wang, Yu-Chieh Lee, Yu-Peng Liu, Chi-Long Chen, Michael Hsiao

**Affiliations:** ^1^ Genomics Research Center, Academia Sinica, Taipei, Taiwan; ^2^ Graduate Institute of Clinical Medicine, College of Medicine, Taipei Medical University, Taipei, Taiwan; ^3^ Division of Hematology and Oncology, Department of Medicine, Taipei Veterans General Hospital, Taipei, Taiwan; ^4^ Graduate Program of Molecular Pharmacology and Toxicology, School of Pharmacy, University of Southern California, Los Angeles, USA; ^5^ Graduate Institute of Medical Sciences, Taipei Medical University, Taipei, Taiwan; ^6^ Department of Genome Medicine, Kaohsiung Medical University, Kaohsiung, Taiwan; ^7^ Department of Pathology, Taipei Medical University Hospital, Taipei Medical University, Taipei, Taiwan; ^8^ Department of Pathology, College of Medicine, Taipei Medical University, Taipei, Taiwan

**Keywords:** DDX3, colorectal cancer, prognosis, metastasis, E-cadherin

## Abstract

**Background:**

Conflicting results regarding the role of DEAD-box polypeptide 3 (DDX3) are seen not only between cancer types but also within the same type of cancer. In this study, we aimed at clarifying the prognostic significance of DDX3 in patients of major cancer types through large cohort survival analysis and further investigated its effects on cancer progression.

**Methods:**

Large cohort survival analysis of 7 cancer types, including colorectal cancer, breast cancer, lung cancer, head and neck cancer, liver cancer, glioblastoma, and ovarian cancer, was performed using public database at RNA level and was further confirmed by IHC analysis at protein level. Phenotype parameters of DDX3 knockdown colon cancer cells and the mechanism of DDX3 regulated cancer progression were investigated *in vitro* and *in vivo*.

**Results:**

In large cohort survival analysis, DDX3 had a significant prognostic predictive power in colorectal cancer at both RNA and protein level. Patients with low DDX3 expression had poor prognosis and frequent distant metastasis. Knockdown of DDX3 enhanced the migration and invasion abilities of colon cancer cells and promoted tumor metastasis *in vivo*. Snail upregulation with decreased membranous E-cadherin expression and reduced cell aggregation were found after DDX3 downregulation.

**Conclusions:**

Our study revealed the strong prognostic effect of DDX3 on colorectal cancer among seven major cancer types through larger cohort survival analysis at RNA and protein level. Low DDX3 expression promotes Snail/E-cadherin pathway mediated cancer metastasis and poor clinical outcome in colorectal cancer patients.

## INTRODUCTION

DEAD box RNA helicases plays an important role in RNA metabolism. DEAD-box polypeptide 3 (DDX3), as a member of the DEAD-box family of RNA helicases, has been reported to participate in pre-mRNA splicing [1], RNA export [2], transcription [3], and translation [4-6]. Considering its complicate function in RNA metabolism, DDX3 has gained increasing attentions for its role in various cancer types and modulates cancer progression in a complex manner. Moreover, this complexity is further increased due to DEAD box proteins generally do not function alone but instead act as a component of multi-protein complexes [7]. The exact function of DDX3 is affected by its interacting partners and is tumor- and/or context-dependent [8]. Both tumor promoting and tumor suppressing effects of DDX3 have been reported. Overexpression of DDX3 has been shown to drive cancer progression in breast cancer through inducing an epithelial-mesenchymal transition like transformation [9]. In glioblastoma, DDX3 promoted cancer cell motility by supporting the expression level of Snail [10]. In gallbladder cancer, high DDX3 expression correlated with poor prognosis, higher pathological stage, and lymph node metastasis [11]. On the contrary, loss of DDX3 expression was demonstrated to be correlated with poor prognosis in lung cancer and oral cancer [12-14].

Interestingly, except for the phenomenon that DDX3 plays different roles in different cancer types, both oncogenic and tumor suppressor functions are also reported in the same kind of cancer. In hepatocellular carcinoma, overexpression of DDX3 was observed in hepatocellular carcinoma and DDX3 was identified as a cellular transforming gene in hepatocarcinogenesis [15]. In contrast, other studies showed loss of DDX3 expression in hepatocellular carcinoma tissue compared to adjacent non-tumor-tissue and tumor growth resulted from DDX3 deregulation in hepatitis virus-associated hepatocellular carcinoma [3, 16]. Therefore, the conflicting roles of DDX3 not only lie between cancer types but also remain inconsistent within the same type of cancer. These controversial results emphasize the urgent need to clarify the prognostic value of DDX3 in major human cancer types.

In this study, we aimed at investigating the prognosis impact of DDX3 through large cohort survival analysis. Our results showed DDX3 has a significant prognostic effect on colorectal cancer among 7 cancer types at either RNA or protein level. Patients with low DDX3 expression level had poor prognosis and more frequent distant metastasis. Downregulation of DDX3 promoted invasion and migration of colon cancer cells and tumor metastasis *in vivo* through Snail/E-cadherin mediated pathway.

## RESULTS

### DDX3 had a significant prognostic predictive value in colorectal cancer in both RNA sequencing and microarray analysis among 7 cancer types

In order to clarify the prognostic value of DDX3 gene expression in human cancers, we performed large cohort survival analysis from two public database with transcriptome analysis results (Supplementary Figure 1). RNA sequencing data from The Cancer Genome Atlas (TCGA) database at UCSC Cancer Genomics Browser (https://genome-cancer.ucsc.edu/) includes the results of 396 colorectal cancer patients, 1099 breast cancer patients, 927 lung cancer patients, 472 head and neck cancer patients, 211 liver cancer patients, 165 glioblastoma patients, and 416 ovarian cancer patients [17]. Survival analysis of RNA microarray database from SurvExpress web resource (http://bioinformatica.mty.itesm.mx/SurvExpress) was assessed on the cohort with the largest case numbers of each common cancer type [18]. There are 482 colorectal cancer patients, 198 breast cancer patients, 1044 lung cancer patients, and 755 ovarian cancer patients from Survexpress Metabase which combines multiple GEO datasets; 538 glioblastoma cases and 283 head and neck cancer cases from TCGA cohort; 162 cases of hepatocellular carcinoma from GEO dataset GSE10143.

**Figure 1:**
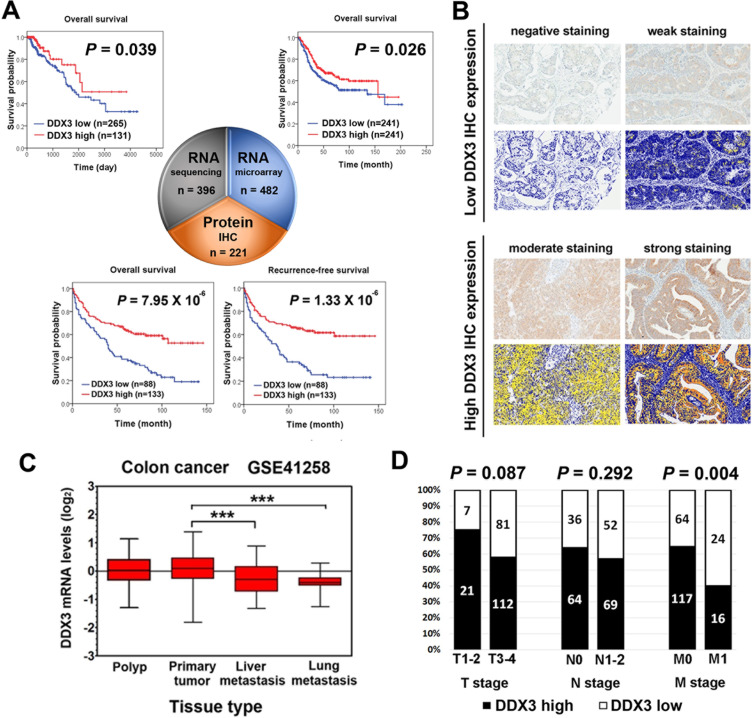
Significant prognostic value of DDX3 in colorectal cancer patients **A.** Kaplan-Meier plots for high versus low DDX3 RNA and protein expression in colorectal cancer. Low DDX3 RNA expression level was correlated with poor prognosis in both RNA sequencing analysis from TCGA database (*P* = 0.039) and RNA microarray analysis from SurvExpress database (*P* = 0.026). Patients with low DDX3 immunoexpression also displayed a significant poor prognosis than those with high DDX3 immunoexpression in overall survival (*P* = 7.95 × 10^−6^) and recurrence-free survival (*P* = 1.33 × 10^−6^). **B.** Representative images of the intensity of DDX3 immunohistochemical staining and corresponding pictures of automated image analysis. Negative and weak IHC staining were classified as low DDX3 expression. Moderate and strong IHC staining were grouped together as high DDX3 expression. Photographs were taken at a magnification of 400×. **C.** DDX3 expression levels in GSE41258 dataset which includes tissues range from polyp, primary colorectal cancer, liver metastasis, and lung metastasis revealed that lower DDX3 expression level were observed in tissue of liver and lung metastatic colorectal cancer (***P < 0.001) compared to primary colorectal cancer tissue. **D.** In clinicopathological analysis, low DDX3 protein expression was significantly associated with the presence of distant metastasis (*P* = 0.004).

The results showed that DDX3 is a significant prognostic predictive indicator for colorectal cancer in both RNA sequencing (hazard ratio [HR] = 0.53, *P* = 0.039) and RNA microarray analysis (HR = 0.72, *P* = 0.026), and patients with low DDX3 expression had poor prognosis (Figure 1A and Supplementary Figure 1). DDX3 is also a significant predictor of prognosis for breast cancer in RNA sequencing analysis (HR = 2.06, *P* < 0.001). Unlike in colorectal cancer, breast cancer patients with high DDX3-RNA level had poor prognosis. But this trend was not seen in RNA microarray analysis (HR = 0.74, *P* = 0.088) (Supplementary Figure 1). In head and neck cancer, low DDX3 expression level was correlated with poor prognosis in RNA microarray analysis (HR = 0.62, *P* = 0.010) but not in RNA sequencing analysis (HR = 1.15, *P* = 0.367) (Supplementary Figure 1). No association between DDX3 expression and survival were seen in lung cancer, liver cancer, glioblastoma, and ovarian cancer (Supplementary Figure 1).

### Colorectal cancer patients with low DDX3 protein expression had poor prognosis and more frequent distant metastasis

Through large cohort survival analysis of DDX3 at transcriptome level, we identified that DDX3 RNA level could predict prognosis in colorectal cancer, breast cancer, and head and neck cancer in either or both of RNA sequencing analysis and RNA microarray analysis. To further elucidate the prognostic value of DDX3, we then analyzed the correlation between DDX3 protein level and patient outcome in these three cancer types by IHC staining on tissue microarray (Supplementary Tables 1-3). All cases were divided into two groups according to DDX3 IHC expression level. Representative images of high and low DDX3 IHC expression and corresponding pictures of automated image analysis were shown in Figure 1B. Survival analysis results revealed that colorectal cancer patients with low DDX3 protein expression exhibited poor overall survival (OS) (*P* = 7.95 × 10^−6^) and recurrence-free survival (RFS) (*P* = 1.33 × 10^−6^) (Figure 1A). In multivariate analysis, only DDX3 expression HR = 1.915, *P* = 0.001 for OS; HR = 2.095, *P* < 0.001 for RFS) and M stage remained independently prognostic (Supplementary Table 4). In contrast, DDX3 protein expression did not have prognostic relevance in breast cancer and head and neck cancer (Supplementary Figure 2). The findings that DDX3 has significant prognostic predictive value in colorectal cancer at transcriptome level in RNA sequencing and RNA microarray analysis and at protein level in IHC analysis strongly indicated the need of further investigation to decipher the role of DDX3 in colorectal cancer.

Therefore, to clarify the clinicopathological role of DDX3 in colorectal cancer, we analyzed a GEO public dataset, GSE41258, which includes tissues range from polyp, primary colorectal cancer, liver metastasis, and to lung metastasis [19]. Lower DDX3 RNA level were observed in tissue of liver and lung metastatic colorectal cancer compared to primary colorectal cancer tissue (Figure 1C). At protein level, low DDX3 IHC expression was also significantly associated with distant metastasis (*P* = 0.004) and tumor recurrence (*P* < 0.001) (Figure 1D and Supplementary Table 5). These result implied that DDX3 dysregulation may have a role in cancer progression through promoting tumor metastasis.

### Repression of DDX3 expression resulted in increased *in vitro* migration and invasion of colon cancer cells and enhanced tumor metastasis *in vivo*

**Figure 2:**
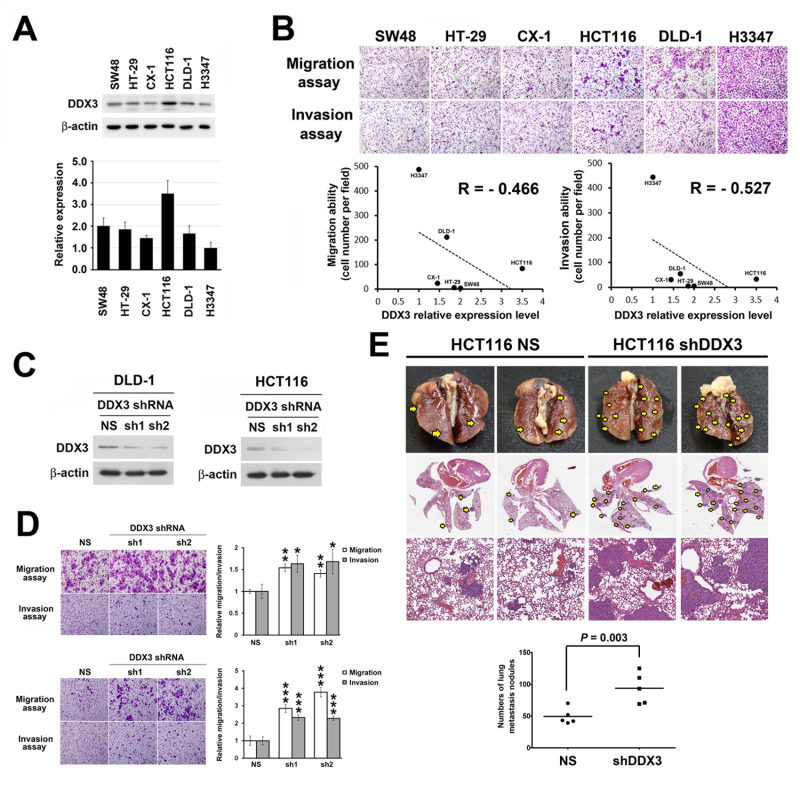
Repression of DDX3 expression resulted in increased cell migration and invasion in colon cancer cells and enhanced tumor metastasis *in vivo* **A.** DDX3 protein expression levels of respective colon cancer cell lines was determined by Western blot (upper panel) and the relative DDX3 expression levels were quantified from triplicate experiments (lower panel). **B.** Migration and invasion abilities of respective colon cancer cell lines were evaluated by transwell assay. Cells were incubated for 10 hours for migration assay in transwell followed by fixing and cell counting. For invasion assay, cells were incubated for 20 hours in transwells pre-coated with 35 μl matrix matrigel. Negative correlation were seen between endogenous DDX3 expression and the migration and invasion abilities of respective colon cancer cell lines. **C.** Western blot results of DDX3 knockdown in DLD-1 cells and HCT116 cells. **D**. DLD-1 cells and HCT116 cells migration and invasion abilities were increased after DDX3 knockdown. For migration assay cells were incubated for 9 hours in transwell followed by fixing and cell counting. Cell invasion potentials were examined using 35 μl matrix matrigel pre-coated transwell after incubation of 14 hours for DLD-1 and 18 hours for HCT116 group. Respective quantification was shown in right panel. * represents *P* < 0.05.** represents *P* < 0.01. *** represents *P* < 0.001. **E.** *In vivo* animal model experiments were performed by injecting DDX3 knockdown HCT116 cells into NSG mice via the tail vein to show the effect of DDX3 on tumor metastasis. Representative gross and H&E staining pictures showed increase of lung metastasis nodules in the DDX3 knockdown group compared with the corresponding non-silenced control cell-injected groups.

Since there is a significant correlation between low DDX3 expression level and metastasis, we then performed *in vitro* migration and invasion assay to further investigate the influence of DDX3 on cancer cell motility and invasive properties. First, we examined the expression level of DDX3 in 6 colon cancer cell lines (Figure 2A). Then, we correlated the endogenous DDX3 expression of these 6 colon cancer cell lines with their invasion and migration abilities. The results showed a negative correlation trend between endogenous DDX3 expression and migration (R = −0.466) and invasion (R = −0.527) abilities in transwell assay (Figure 2B). Cell lines with lower DDX3 expression such as DLD-1 and H3347 tended to have higher migration/invasion ability. To further explore the functional effects of DDX3 knockdown on colon cancer metastasis, DDX3 level was suppressed using 2 DDX3 specific shRNAs in DLD-1 and HCT116 cells, respectively (Figure 2C). Increased cell migration and invasion abilities were observed in DDX3 knockdown DLD-1 cells and HCT116 cells (Figure 2D) in transwell assay. In the proliferation assay, no significant difference was seen between the proliferation activity of DDX3 knockdown group and control group in short-term culture (Supplementary Figure 3). *In vivo* animal model experiments were also performed to examine the effect of DDX3 on tumor metastasis. HCT116 cells with DDX3 knockdown were injected into NSG mice via the tail vein. Increased lung metastasis nodules was observed in the DDX3 knockdown group compared with the corresponding non-silenced control cell-injected groups (*P* = 0.003, Figure 2E). The findings indicated the potential anti-metastasis function of DDX3 in colon cancer.

### DDX3 regulated cell migration and invasion via Snail/E-cadherin mediated pathway

**Figure 3:**
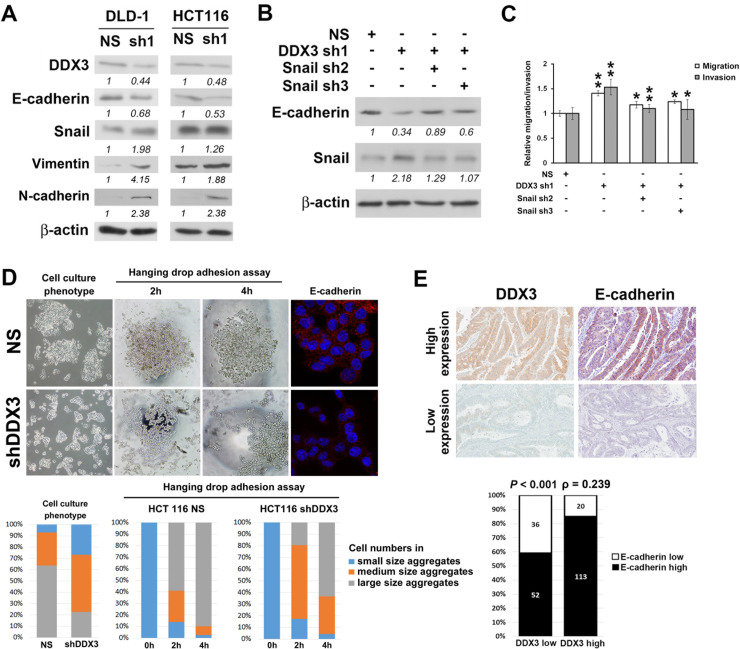
DDX3 regulated cell migration and invasion via E-cadherin/Snail signaling pathway **A.** Upregulation of transcriptional repressor Snail and decrease of E-cadherin expression and increase of vimentin and N-cadherin expression were found in DLD-1 and HCT116 cells after DDX3 knockdown. **B.** Western blot results of DDX3 knockdown DLD-1 cells after Snail repression. **C.** Snail repression significantly decreased migration and invasion potentials of DDX3 knockdown cells. * represents *P* < 0.05. ** represents *P* < 0.01. **D.** In HCT116 cells, knockdown of DDX3 reduced the cell numbers in large size cell aggregation. In hanging drop adhesion assay, decrease of cell numbers in large size cell aggregation were also seen in DDX3 knockdown group. Loss of E-cadherin membrane staining was observed in DDX3 knockdown HCT116 cells compared to HCT116 non-silenced control cells which expressed strong E-cadherin membrane staining. **E.** Correlation analysis showed a positive correlation between IHC expression of DDX3 and E-cadherin (Spearman's ρ = 0.239, *P* < 0.001). Representative images of 2 colorectal adenocarcinoma cases, one with high DDX3 and E-cadherin IHC expression, the other with low DDX3 and E-cadherin IHC expression.

To dissect the molecular mechanism of DDX3-mediated cell migration and invasion in colon cancer, we evaluated the expression difference of several epithelial-mesenchymal transition markers between DDX3 knockdown cells and control cells. Upregulation of transcriptional repressor Snail and decrease of E-cadherin expression and increase of vimentin and N-cadherin expression were found in DLD-1 and HCT116 cells after DDX3 reduction (Figure 3A). We then repressed Snail level in DLD-1 cells with DDX3 knockdown using Snail shRNA (Figure 3B). Knockdown of Snail significantly reduced migration and invasion potentials of DDX3 knockdown cells (Figure 3C). We also observed that, in HCT116 cells, a cell line grows with high degree of cell-cell attachment, knockdown of DDX3 reduced the cell numbers in large size cell aggregation (Figure 3D). In the hanging drop adhesion assay, decrease of cell numbers in large size cell aggregation were also seen in DDX3 knockdown group. (Figure 3D). Immunofluorescence staining of E-cadherin was performed to confirm the decrease of E-cadherin expression in DDX3 knockdown cells found in western blot analysis. Loss of E-cadherin membrane staining was seen in DDX3 knockdown HCT116 cells compared to HCT116 non-silenced control cells which expressed strong E-cadherin membrane staining (Figure 3D). The negative control staining of E-cadherin on HCT116 non-silenced control cells showed negative immunoexpression (Supplementary Figure 4). In clinical colorectal-adenocarcinoma specimens, significant positive correlation was also observed between DDX3 and E-cadherin IHC expression (Spearman's ρ = 0.239, *P* < 0.001) (Figure 3E). Images of 2 representative colorectal adenocarcinoma cases, one with high DDX3 and E-cadherin expression, the other with low DDX3 and E-cadherin expression were also shown. These results implicated that DDX3 regulated cell migration and invasion through Snail/E-cadherin mediated pathway in colorectal cancer.

## DISCUSSION

In present study, we revealed that DDX3 has strong prognostic impact on colorectal cancer patients compared to other major cancer types through a large cohort screening of the public database at RNA level and confirming the finding by IHC analysis at protein level. Growing numbers of public datasets allow us to determine the influence of the target marker in broad population and sever as a good method to overcome the conflicting results regarding the role of the marker. Surprisingly, in large cohort survival analysis of our study, DDX3 did not play a role in patient outcomes of lung cancer, hepatocellular carcinoma, and gliobalstoma which DDX3 has previously been reported to be an important tumor progression regulator [9, 10, 13, 16]. Only in colorectal cancer, low DDX3 expression had predictive power for poor prognosis in both RNA sequencing and RNA microarray analysis. One of the possible explanation of these conflicts is that, in previous studies, DDX3 regulated tumor progression was mainly found in virus-associated cancer such as hepatitis B virus (HBV) or hepatitis C virus (HCV) associated hepatocellular carcinoma and human papillomavirus (HPV) associated lung cancer [12, 16]. Therefore, in these cancer types, DDX3 may significantly modulate tumor progression in specific patient group but loss its role in other patients. Our study emphasized that large cohort survival analysis using public database could give us a complete view of the role of certain marker in clinical patients and increase the future application possibility of therapeutic decision making or drug development. However, it is worth mentioning the discrepancy of the results between RNA sequencing and RNA microarray analysis noted in our study. In breast cancer and head and neck cancer, DDX3 RNA level has a prognostic significance in either RNA sequencing or RNA microarray analysis, but not in both. This kind of inconsistency may result from highly dynamic and complex regulation of RNA in living cells and the easy degradation of RNA during extraction and storage [20, 21] and therefore implies the importance to confirm the results at protein level as in our study. Another interesting finding to note is that, in breast cancer, in spite of failing to associate with patient outcome in RNA microarray analysis and IHC analysis, high DDX3 level is a poor prognostic indicator in RNA sequencing analysis. The positive correlation between DDX3 expression level and poor survival is a result opposite to the trend shown in colorectal cancer and head and neck cancer. The poor prognostic impact of DDX3 RNA level on breast cancer is generally compatible with previous studies which indicated that DDX3 overexpression promoted tumor progression through inducing epithelial-mesenchymal transition like transformation [9]. Hence, the contradictory prognosis effect of DDX3 on different cancer types revealed in present study also reflects its double-edged role and the underlying complex mechanism in cancer cells.

As its complicated role, the mechanism of DDX3 dysregulation also varies according to cancer types. In breast cancer, DDX3 was reported as a hypoxia inducible factor-1 inducible gene and DDX3 expression correlated with HIF-1α related proteins expression [22, 23]. On the other hand, DDX3 has a central place in virus-associated cancer for its well-known function in virus lifecycle [24-27]. Hepatitis virus mediated DDX3 dysregulation was shown to be involved in carcinogenesis of hepatitis virus-associated hepatocellular carcinoma[16, 28]. In lung cancer, p53 inactivation by either HPV E6 oncoprotein or p53 mutation reduced DDX3 transcription [12, 13]. Furthermore, DDX3 also helped regulating p53 stabilization and DNA damage-induced intrinsic apoptosis in wild-type p53 cells [29]. The close interactions between DDX3 and p53 and frequent p53 mutation in colorectal cancer may be the reasonable explanation for the strong prognostic predictive ability of DDX3 in colorectal cancer [30, 31]. Further investigation is therefore necessary to clarify the relation between DDX3 expression and p53 in colorectal cancer.

Our study depicted that DDX3 deregulation promotes colorectal cancer progression through enhancing cell invasion and migration abilities by Snail/E-cadherin pathway. Previous studies also demonstrated the alteration of cell motility and invasion regulated by DDX3 in different cancer types. Loss of DDX3 expression promoted tumor progression through MDM2/Slug/E-cadherin pathway in lung cancer [13]. In breast cancer, which DDX3 has an oncogenic role, DDX3 overexpression increased cell invasion and motility by an epithelial-mesenchymal transition like change in cell morphology with decrease of E-cadherin expression [9]. As one of the important marker in epithelial-mesenchymal transition, Snail was also shown to be regulated by DDX3 and then increased cell migration in glioblastoma [10]. A recent research pointed out that knockdown of DDX3 increased cell adhesion and decreased cell migration through Rac1-mediated pathway [32]. In the same study, the expression of Wnt/β-catenin target genes were also influenced by DDX3-Rac1 signaling and further enhanced cancer metastasis [32]. Considering that Wnt/β-catenin signaling axis is critical for tumorigenesis of colorectal cancer, whether DDX3 could mediate Wnt/β-catenin pathway in colorectal cancer is worth of future research.

In conclusion, our results highlighted the strong prognostic value of DDX3 in colorectal cancer from 7 major cancer types through large cohort survival analysis of DDX3 at transcriptome and protein level. Patients with low DDX3 expression had poor prognosis and more frequent distant metastasis. Downregulation of DDX3 enhanced tumor progression through Snail/E-cadherin pathway mediated cell migration and invasion. Our study revealed that DDX3 is a usefu l prognostic marker and may be a promising therapeutic target in colorectal cancer.

## MATERIALS AND METHODS

### Online database survival analysis

Large cohort survival analysis was performed by using two public database with transcriptome analysis data. RNA sequencing data of 3686 patients of 7 cancer types including colorectal cancer, breast cancer, lung cancer, head and neck cancer, liver cancer, glioblastoma, and ovarian cancer were obtained from The Cancer Genome Atlas (TCGA) database at UCSC Cancer Genomics Browser (https://genome-cancer.ucsc.edu/) [17]. For survival analysis, the samples of each cancer types were divided into three groups of identical size of high, middle and low expression levels according to the built-in setup of the website. The group with high RNA expression was compared to the cohort combined the group with middle and low RNA expression.

RNA microarray analysis results of 3462 patients of these 7 cancer types were collected from SurvExpress web resource (http://bioinformatica.mty.itesm.mx/SurvExpress) which includes datasets from Gene Expression Omnibus (GEO) and TCGA database [18]. Average expression level of all DDX3 probes with quantile-normalization were used to represent the RNA expression level of DDX3 in each dataset. The samples were split into two risk groups of the same size depending on the prognostic index estimated by beta coefficients multiplied by gene expression values. Overall survival was used as the end point of statistical analysis for both RNA sequencing and RNA microarray data of all cancer types.

### Patients

Formalin-fixed paraffin embedded (FFPE) tissues of 221 colorectal cancer patients, 152 breast cancer patients, and 107 head and neck cancer patients were obtained from the archive of Cancer Tissue Core of the Taipei Medicine University associated Hospital (Taipei, Taiwan) from 1998 to 2006 (Supplementary Tables 1-3). Patients who received preoperative chemotherapy or radiation therapy or who received incomplete surgical resection were excluded. All colorectal cancer patients received surgical resection and stage III and IV patients and stage II patients with high risk underwent adjuvant chemotherapy including 5-fluorouracil (5-FU)/leucovorin (LV), Capecitabine (Xeloda), or tegafur-uracil (UFUR) plus leucovorin (LV). For breast cancer cohort, patients were treated with either modified radical mastectomy or partial mastectomy with axillary lymph node dissection, and then adjuvant chemotherapy and/or hormone therapy according to hospital guideline. Head and neck cancer patients underwent adjuvant chemotherapy and/or radiotherapy after surgical treatment. Overall survival (OS) and recurrence-free survival (RFS) were defined as the interval from surgery to death from any cause and recurrence or distant metastasis or death, respectively. All cases were staged according to the caner staging manual of the American Joint Committee on Cancer and the histological cancer type was classified according to World Health Organization classification. The study was carried out with the approval of the Institutional Review Boards and the permission from the ethics committees of Taipei Medical University (IRB-99049).

### Immunohistochemistry staining and interpretation

Representative 1-mm-diameter cores of each tumor from the formalin-fixed paraffin embedded tissues were selected by morphology typical of the diagnosis for tissue microarray construction. Immunohistochemical (IHC) staining was performed on 5-micrometer-thick tissue sections cut from the tissue microarray using an automated immunostainer (Ventana Discovery XT autostainer, USA). Briefly, sections were first dewaxed in a 60°C oven, deparaffinized in xylene, and rehydrated in graded alcohol. Antigens were retrieved by heat induced antigen retrieval for 30 minutes with TRIS-EDTA buffer. Slides were stained with a polyclonal rabbit DDX3 antibody (1:75; Sigma-Aldrich, USA) or a monoclonal mouse E-cadherin antibody (1:40; DAKO, USA). The sections were subsequently counterstained with hematoxylin, dyhydrated, and mounted. The IHC staining assessment was independently conducted by 2 pathologists (Chia-Yi Su and Michael Hsiao). DDX3 IHC expression was scored according to previous paper [16]. In brief, a four-tiered approach was used for cytoplasmic DDX3 IHC expression in tumor cells: negative staining, weak staining, moderate staining, and strong staining. Negative staining and weak staining were defined as low DDX3 IHC expression and moderate to strong staining were defined as high DDX3 IHC expression. For E-cadherin IHC staining interpretation, only membrane staining was assessed. E-cadherin IHC staining was considered as high expression when > 50% of cancer cells had a membranous staining and cases with ≤ 50% tumor cells with membranous staining were considered as low expression [33]. An automated image analysis system (Aperio Technology, USA) was used to validate the results after slides were scanned with a ScanScope XT Slide Scanner (Aperio Technologies, USA). Quantification of immunohistochemical staining was analyzed by color translation and automated thresholding algorithm. For DDX3 IHC staining, over 50% tumor area with medium (yellow color) or strong staining (orange color) was identified as high DDX3 IHC expression by automated image analysis system. The results from automated image analysis system were compared with the IHC expression status interpreted by pathologists. E-cadherin IHC staining was also assessed by Aperio Membrane v9 Algorithm and compared with the results interpreted by pathologists.

### Cell culture

Human colon cancer cell line CX-1, DLD-1, H3347 cells were maintained in RPMI 1640 medium. HCT116 and HT-29 cells were maintained in McCoy's 5A modified medium (Sigma, St. Louis, MO, USA). SW48 cells were cultured in Leibovitz L-15 medium (Sigma, St. Louis, MO, USA). Mediums were all supplemented with 10% fetal bovine serum (GIBCO, Grand Island, NY, USA), penicillin (100 unit/ml), and streptomycin (100 μg/ml). Cells were incubated in 95% air, 5% CO_2_ humidified atmosphere at 37 °C.

### Lentiviral-based shRNA production and infection

The lentiviral shRNA constructs were purchased from Thermo Scientific (Pittsburgh, PA, USA). Lentiviruses were produced by co-transfection of shRNA-expressing plasmid, envelope plasmid (pMD.G) and packaging plasmid (pCMV-dR8.91) in 293T cells using calcium phosphate (Invitrogen, Carlsbad, CA, USA). The 293T cells were incubated for 18 h, and the culture medium was then removed, and refreshed. The viral supernatants were harvested and tittered at 48 and 72 h post-transfection. Monolayer cells were infected with the lentiviruses in the presence of polybrene, and were further selected using puromycin.

### Western blot analysis

Cells were lysed at 4 °C in RIPA buffer supplemented with protease and phosphatase inhibitors. Equal amounts of proteins were separated by SDS-polyacrylamide gels, and then transferred to PVDF membrane (Millipore, Bedford, MA, USA). After blocking with 5% non-fat milk, the membrane was reacted with specific antibodies overnight at 4 °C and then incubated with horseradish peroxidase conjugated secondary antibody for 1 h. The blots were visualized using ECL-Plus detection kit (PerkinElmer Life Sciences, Boston, MA, USA).

### Proliferation assay and migration and invasion assay

Cell proliferation was assessed by trypan blue exclusion assay for 120 hours with a cell density of 1×10^4^/well seeded. The *in vitro* migration and invasion ability were assessed using Transwell assay (Millipore, Bedford, MA, USA). For invasion assay, transwell was additional pre-coated with 35 μl of 3X diluted matrix matrigel (Bd Biosciences Pharmingen, San Diego, CA, USA) for 30 minutes. Cells of 2 × 10^5^ in serum-free culture medium were added to the upper chamber of the device, and the lower chamber was filled with 10% FBS culture medium. After indicated hours of incubation, upper surface of the filter was carefully removed with a cotton swab. The filter was then fixed, stained and photographed. Cells of migration and invasion were quantified by counting the cells in three random fields per filter.

### Cell aggregation assay

After growing to 80% confluence, cells were trypsinized and resupended at a concentration of 1.5×10^6^ cells per ml in medium with FBS and then seeded in 10 cm Petri dishes. Cell aggregates were photographed and evaluated after 24 hrs of incubation. For the hanging drop adhesion assay, cells were trypsinzied and resuspended at 2.5×10^5^ cells per ml in culture medium, and 20ul drops of cell suspension were placed on the inside surface of 10 cm Petri dishes lids. After trituration of each drop by a 20ul pipette for 10 times, the lids were inverted and placed on dishes filed with 10ml culture medium. Cell aggregation status in three random fields were photographed and calculated. Cell aggregates were classified as: small aggregates, 0-10 cells; medium aggregates, 10-40 cells; large aggregates, >40 cells. The number of cells in each size of cell aggregation were counted.

### Immunofluorescence staining and confocal microscopy

The cells were incubated with E-cadherin antibody (# 3195s, 1:100; Cell Signaling Technology, USA) at 4°C overnight and incubated with goat anti-rabbit IgG (H+L) Alexa Fluor® 594 secondary antibody (A11012. 1:200: Invitrogen. USA) for 1 hr at room temperature, and then plated onto slides and analyzed with a spin-disk confocal microscopy (Molecular Devices, USA) and Metamorph software.

### Animal study

All animal experiments were conducted in accordance with a protocol approved by the Academia Sinica Institutional Animal Care and Utilization Committee. Age-matched male NSG mice (6 to 8 weeks of age) were used. To evaluate metastasis status, 1 × 10^6^ cells were resuspended in 0.1 ml of PBS and injected into the lateral tail vein (n=5). Metastatic lung nodules were counted and were further confirmed via H&E staining under microscope.

### Statistical analysis

Survival analyses were generated using univariate and multivariate Cox proportional hazards regression and Kaplan–Meier method. The association between DDX3 immunoreactivity and clinicopathological characteristics wERE analyzed by Chi-Square test. Spearman's rank correlation analysis was used to determine the correlation of the IHC expression between DDX3 and E-cadherin. For clinical data analysis, a *P*-value of less than 0.05 was considered as statistically significant. Student's *t*-test was used to compare DDX3 RNA expression levels between different tissue types. Pearson correlation analysis was used to examine the correlation between the relative endogenous DDX3 protein expression and the migration and invasion abilities in colon cancer cell lines. Student's *t*-test was also used to compare the results of migration and invasion assay and *in vivo* metastasis experiment. The *P* values with the following levels were considered significant: * *P*<0.05, ** *P*<0.01, *** *P*<0.001. Statistics analysis was performed on SPSS 17.0 software (SPSS, USA).

## SUPPLEMENTARY MATERIAL FIGURES AND TABLES


