# Supplementary material for: DDX3 as a strongest prognosis marker and its downregulation promotes metastasis in colorectal cancer
Source: Oncotarget. 2015 Jun 2;6(21):18602–12. doi: 10.18632/oncotarget.4329 (PMC4621913; doi:10.18632/oncotarget.4329)
Supplement: Supplementary file 1 [file oncotarget-06-18602-s001.pdf]

DDX3 as a strongest prognosis marker and its downregulation promotes metastasis in colorectal cancer

Supplementary Material

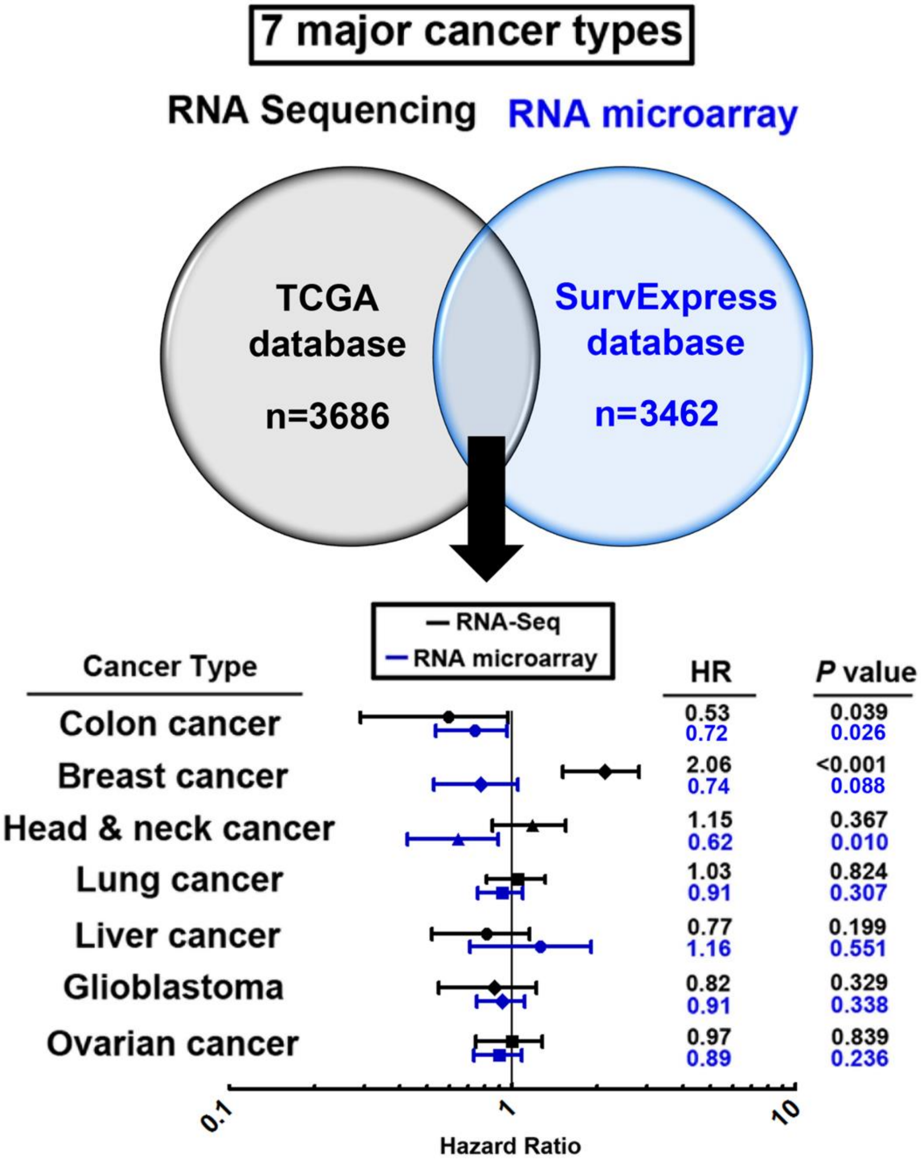

Supplementary Figure 1: Survival analysis of RNA sequencing data from TCGA cohort and RNA microarray results from SurvExpress web resource showed that DDX3 is a significant prognostic predictive indicator in colorectal cancer in both RNA sequencing analysis (hazard ratio [HR] = 0.53, P = 0.039) and RNA microarray analysis (HR = 0.72, P = 0.026), and patients with low DDX3 expression had poor prognosis. In contrast, high DDX3 RNA level was correlated with prognosis for breast cancer in RNA sequencing analysis (HR = 2.06, P < 0.001), but this trend was not seen in RNA microarray analysis. In head and neck cancer, low DDX3 expression level was associated with poor prognosis in RNA microarray analysis (HR = 0.62, P = 0.010) but not in RNA sequencing analysis. No association between DDX3 RNA expression and survival were seen in lung cancer, liver cancer, glioblastoma, and ovarian cancer.

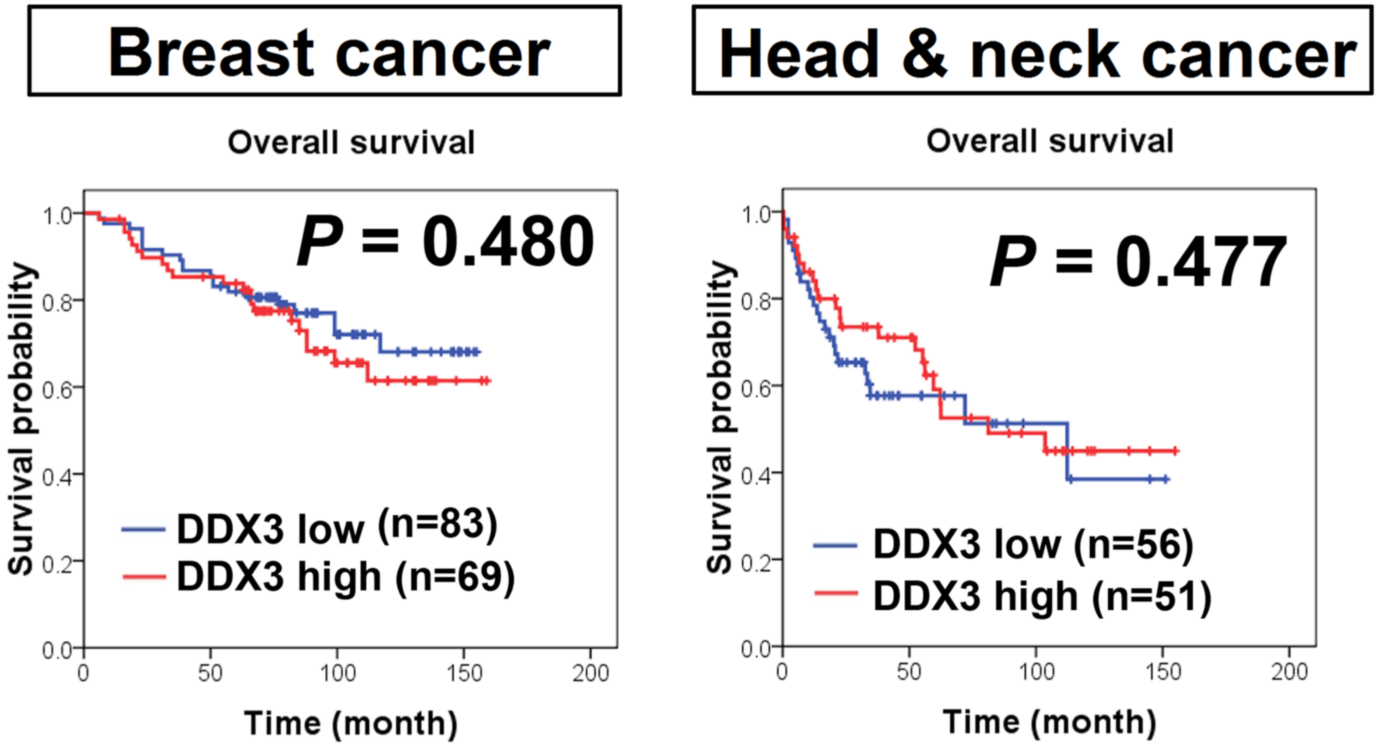

Supplementary Figure 2: Kaplan-Meier plots for high versus low DDX3 protein expression in breast and head and neck cancer. No prognostic significance was seen between DDX3 protein expression and patient outcome in either breast cancer or head and neck cancer.

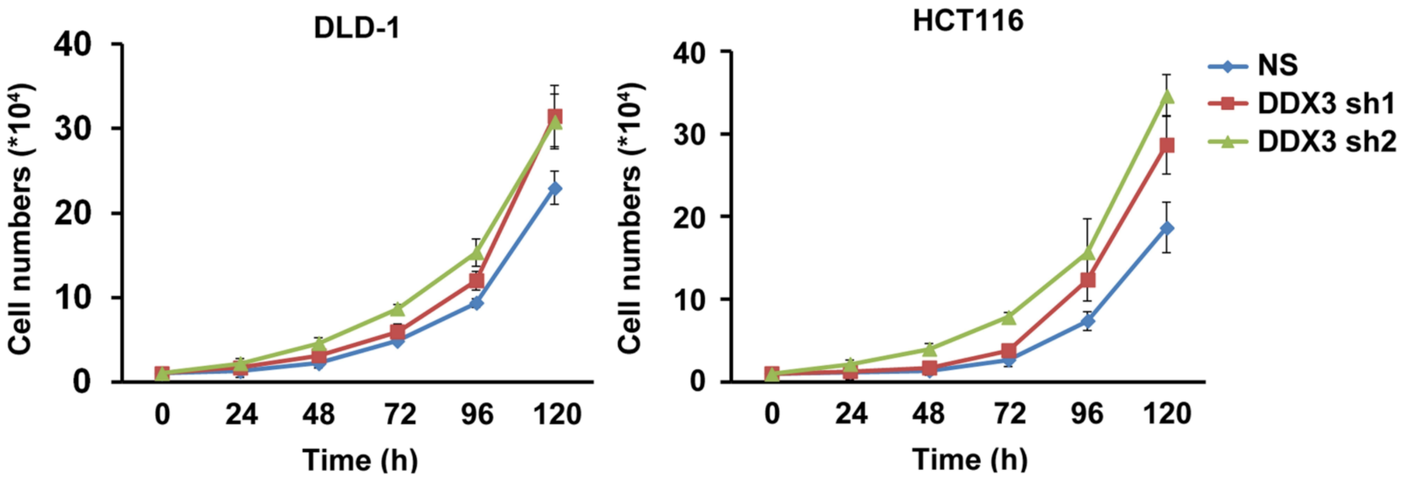

Supplementary Figure 3: In trypan blue exclusion assay, no significant difference was seen between the proliferation activity of DDX3 knockdown group and control group in short-term culture.

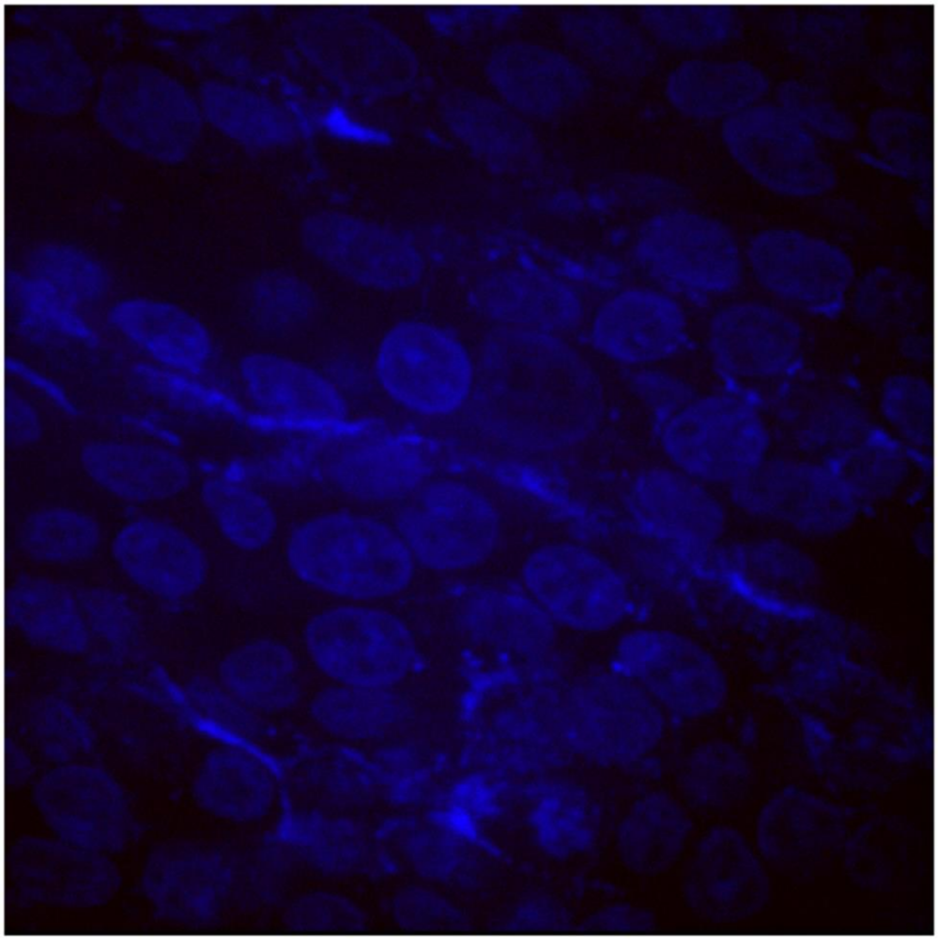

Supplementary Figure 4: Negative control for immunofluorescence staining of E-cadherin on HCT116 non-silenced control cells.

Supplementary table 1. Clinicopathological and demographic characteristics of 221 colorectal cancer patients

Clinicopathological feature	N (%)
All patients	221 (100)
Age	
<65 y	73 (33)
≧65 y	148 (67)
Gender	
Male	127 (57)
Female	94 (43)
Tumor location	
Cecum	8 (4)
Ascending colon	30 (13)
Transverse colon	28 (13)
Descending colon	18 (8)
Rectosigmoid	137 (62)
T stage	
T1	9 (4)
T2	19 (9)
T3	160 (72)
T4a	20 (9)
T4b	13 (6)
N stage	
N0	100 (45)
N1a	36 (16)
N1b	30 (14)
N1c	2 (1)
N2a	28 (13)
N2b	25 (11)
M stage	
M0	181 (82)
M1	40 (18)
Vascular invasion	
No	101 (46)
Yes	120 (54)
Perineural invasion	
No	170 (77)
Yes	51 (23)
Tumor histology	
Nonmucinous	202 (91)
Mucinous	19 (9)
Recurrence	
No	141 (64)
Yes	80 (36)

Supplementary table 2. Clinicopathological and demographic characteristics of 152 breast cancer patients

Clinicopathological feature	N (%)
All patients	152 (100)
Age	
<50 y	71 (47)
≧50 y	81 (53)
Surgical type	
Modified radical mastectomy	59 (39)
Partial mastectomy	93 (61)
ER status	
Positive	87 (57)
Negative	65 (43)
PR status	
Positive	69 (45)
Negative	83 (55)
HER2 status	
Positive	45 (30)
Negative	107 (70)
Recurrence	
No	111 (73)
Yes	41 (27)

Supplementary table 3. Clinicopathological and demographic characteristics of 107 head and neck cancer patients

Clinicopathological feature	N (%)
All patients	107 (100)
Age	
<50 y	53 (50)
≧65 y	54 (50)
Gender	
Male	99 (93)
Female	8 (7)
Tumor location	
Buccal mucosa	48 (45)
Tongue	34 (32)
Gingiva	16 (15)
Lip	3 (3)
Others	6 (5)
Pathological stage	
I	33 (31)
II	21 (20)
III	11 (10)
IV	42 (39)
Lymphovascular invasion	
Positive	36 (34)
Negative	71 (66)
Perineural invasion	
Positive	56 (52)
Negative	51 (48)
Recurrence	
No	43 (40)
Yes	64 (60)

Supplementary Table 4.

Univariate and multivariate analysis of DDX3 expression and clinicopathological parameters in colorectal cancer patients

Variables	Overall survival						Recurrence-free survival					
	Univariate analysis			Multivariate analysis			Univariate analysis			Multivariate analysis		
	HR	95% CI	*P*	HR	95% CI	*P*	HR	95%CI	*P*	HR	95%CI	*P*
T stage												
T1+2	1	1.191-5.	0.015	1	0.910-	0.087	1	1.401-	0.006	1	0.951-	0.065
T3+4	2.441	006		1.907	3.998		3.191	7.268		2.213	5.149	
N stage												
N0	1	0.948-1.	0.094	1	0.905-	0.145	1	1.060-	0.024	1	0.954-	0.083
N1-2	1.367	969		1.338	1.979		1.554	2.278		1.431	2.148	
M stage												
M0	1	4.233-9.	<0.001	1	3.880-	<0.001	1	4.088-	<0.001	1	3.245-	<0.001
M1	6.442	802		6.278	10.160		6.197	9.392		5.228	8.423	
Vascular invasion												
No	1	0.973-2.	0.070	1	0.424-	0.087	1	1.168-	0.006	1	0.466-	0.246
Yes	1.404	027		0.671	1.060		1.722	2.540		0.753	1.216	
Perineural invasion												
No	1	1.085-2.	0.018	1	0.732-	0.554	1	1.339-	0.001	1	0.865-	0.186
Yes	1.621	422		1.145	1.792		1.998	2.979		1.353	2.116	
DDX3 expression												
High	1	1.547-3.	<0.001	1	1.319-	0.001	1	1.677-	<0.001	1	1.417-	<0.001
Low	2.222	190		1.915	2.778		2.443	3.560		2.095	3.096	

OS: overall survival; RFS: recurrence-free survival; HR: hazard ratio; CI: confidence interval

Supplementary Table 5. Correlation of clinicopathological features and DDX3 protein expression in colorectal cancer patients

Clinicopathological feature	n	DDX3 expression, n (%)		*P*
		Low (n=88)	High (n=133)	
Age				
<65 y	73	28 (38.4)	45 (61.6)	0.755
≧65 y	148	60 (40.5)	88 (59.5)	
Gender				
Male	127	51 (40.2)	76 (59.8)	0.905
Female	94	37 (39.4)	57 (60.6)	
T stage				
T1+T2	28	7 (25.0)	21 (75.0)	0.087
T3+T4	193	81 (42.0)	112 (58.0)	
N stage				
N0	100	36 (36.0)	64 (64.0)	0.292
N1+N2	121	52 (43.0)	69 (57.0)	
M stage				
M0	181	64 (35.4)	117 (64.6)	0.004
M1	40	24 (60.0)	16 (40.0)	
Vascular invasion				
No	101	35 (34.7)	66 (65.3)	0.150
Yes	120	53 (44.2)	67 (55.8)	
Perineural invasion				
No	170	66 (38.8)	104 (61.2)	0.581
Yes	51	22 (43.1)	29 (56.9)	
Tumor histology				
Nonmucinous	202	76 (37.6)	126 (62.4)	0.030
Mucinous	19	12 (63.2)	7 (36.8)	
Recurrence				
No	141	41 (29.1)	100 (70.9)	<0.001
Yes	80	47 (58.8)	33 (41.3)	
